# A NISQ Method to Simulate Hermitian Matrix Evolution

**DOI:** 10.3390/e24070899

**Published:** 2022-06-29

**Authors:** Keren Li, Pan Gao

**Affiliations:** 1 Peng Cheng Laboratory, Shenzhen 518055, China; 2 Beijing Academy of Quantum Information Sciences, Beijing 100193, China

**Keywords:** matrix evolution, NISQ, quantum algorithm

## Abstract

As a universal quantum computer requires millions of error-corrected qubits, one of the current goals is to exploit the power of noisy intermediate-scale quantum (NISQ) devices. Based on a NISQ module–layered circuit, we propose a heuristic protocol to simulate Hermitian matrix evolution, which is widely applied as the core for many quantum algorithms. The two embedded methods, with their own advantages, only require shallow circuits and basic quantum gates. Capable to being deployed in near future quantum devices, we hope it provides an experiment-friendly way, contributing to the exploitation of power of current devices.

## 1. Introduction

Building up a large-scale error-corrected quantum computer is to be one of the greatest scientific and engineering achievements [1,2,3,4]. However, stringent requirements such as millions of qubits with high accuracy are far to meet. Preskill coined “Noisy Intermediate-Scale Quantum” (NISQ) to describe this era, where NISQ devices represent the current state of the art in the fabrication of quantum devices [5]. The leading quantum computers contain up to a few hundred physical qubits, but provide rare practical applications as error correction is missing [6,7]. Therefore, while polishing the hardware-related techniques, one present-day goal is to exploit the power of current machines.

Matrix evolution is computationally hard in numerical mathematics, as $O(d^{3})$ operations are required for an unstructured d×d matrix [8]. With advent of quantum algorithms, this can be solved to some extent by instinct priority of fault-tolerant quantum computation on matrix multiplications. For example, complexity of *t*-time analog Hamiltonian evolution is O(t). Furthermore, certain digital algorithms were proposed for hermitian matrices’ evolution, which produce quantum speedups in many scenarios, such as simulation algorithms, quantum principal component analysis, quantum matrix inversion, and their generalizations [9,10,11,12,13,14,15]. However, deep quantum circuits and inaccessible oracles are required, which hinder their applications on near term devices. Accordingly, one question need be addressed: How to realize matrix evolution on current NISQ devices?

NISQ algorithms are a class of algorithms with no explicit requirements for error correction, promising to be deployed on NISQ hardware [5,16]. In regard to matrix evolution, we explore its near future application by introducing one typical NISQ module–layered circuit. Consequently, in Section 2, a heuristic protocol is proposed to employ a layered circuit to simulate hermitian matrix evolution, which is experimental friendly and can be employed in near-term applications of algorithms. To generate proper layered circuits, two methods are embedded in this protocol. The first is inspired by optimal control theory, which finds the simulating circuit directly, but with no scalability. The second, averting the scalability problem, generates the simulating circuit by a hybrid quantum-classical paradigm [17]. Both simulating circuits are with basic quantum gates and a pre-set depth, with the consumption of generating those circuits analyzed in Section 3. To support feasibility, simulating circuits are generated and validated numerically in Section 4, where hermitian matrices are set as density matrix of Bell state, GHZ state, and Hamiltonian of Crotonic acid molecular [18]. Compared with the method such as product formula and density matrix evolution, simulating circuits by our methods are with shallower depth, and more friendly to the experimental realizations. Furthermore, as a generalization of linear combination unitaries and layered circuit [19,20,21], ancillary layered circuit is proposed in our protocol, which not only serves as a quantum compiler here, but also an essential subroutine for NISQ algorithms.

## 2. Result

For hermitian matrices evolution, Lie–Trotter products and density matrix evolution provide solutions. We briefly review them here.

**Figure 1:**
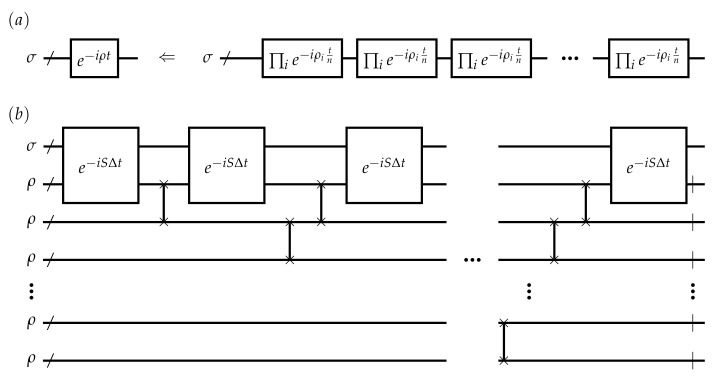
Conventional method to realize $e^{-i\rho t}$, where ρ is a hermitian matrix. (**a**) is via decomposition from Lie–Trotter products and (**b**) is the density matrix evolution using infinitesimal swaps $e^{-iS\Delta t}$. Wherein, /denotes a register of multi qubits, and|means tracing out the corresponding register.

In the first case, for general Hamiltonian $\rho =\sum_{i}\rho_{i}$ with $\rho_{i}$ be local interactions, $e^{-i\rho t}$ can be simulated by the lowest order Lie–Trotter product formula [22],

(1) $$\begin{array}{c} {\left(\prod_{i}e^{-i\rho_{i}\Delta t}\right)}^{n}\to e^{-i\rho t}+{O(||\rho ||}^{2}t^{2}/n), \end{array}$$

with Δt=t/n, as shown in the schematic process in Figure 1a. $n=O(t^{2}\epsilon^{-1}||\rho |{|}^{2})$ denotes the repetition times, and therefore the circuits size and depth for full $e^{-i\rho t}$ with desired accuracy ϵ. Though repeated applications of simulated circuit is feasible in theory, a low-depth quantum circuit is preferred by current NISQ devices as the limited coherence time. Therefore, under the circumstance of a tolerant accuracy, the implementation appears unfriendly to current or near term devices.

In the second case, ρ is not only Hermitian but also positive semi-definite and unit trace, that is, ρ is a physical quantum state, $e^{-i\rho t}$ can be realized by multiple copies of ρ and infinitesimal swap operations [10]. Assuming that σ is another quantum state that $e^{-i\rho t}$ act on, the infinitesimal swap operation has such effect,

(2) $$\begin{array}{c} {\mathrm{tr}}_{p}\left[e^{-iS\Delta t}\rho ⊗\sigma e^{iS\Delta t}\right]=\sigma -i\Delta t\left[\rho ,\sigma \right]+O\left(\Delta t^{2}\right), \end{array}$$

where ${\mathrm{tr}}_{p}$ is the partial trace over ρ and *S* is the swap operator. Shown in Figure 1b, density matrix evolution with respect to σ can be constructed by repeated applications of (2) with n=t/Δt copies of ρ. Therefore, if the swap *S* and its infinitesimal exponential operation $e^{-iS\Delta t}$ can be implemented in a single layer circuit, both size and depth of using Equation (2) are $O(t^{2}\epsilon^{-1})$. For accurate simulation, this depth and size of the circuit still dissatisfy the characteristics of current devices, and thus hinder its near term application.

**Figure 2:**
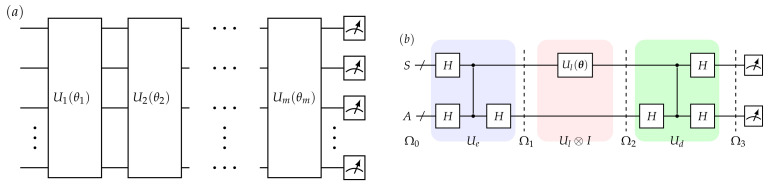
Layered circuit and ancillary layered circuit. (**a**) gives an example of an *m* layered circuit, which is employed in (**b**) as $U_{l}\left(\theta \right)$. $U_{i}\left(\theta_{i}\right)$ are parametrized by tuneable $\theta_{i}$. (**b**) is ancillary layered circuit, which has three parts: encoding circuit (blue) $U_{e}$, layered circuit (pink) $U_{l}⊗I$ and decoding circuit (green) $U_{d}$. $\Omega_{0}$, $\Omega_{1}$, $\Omega_{2}$, and $\Omega_{3}$ represent temporary states.

One typical NISQ module is the layered circuit, which concretely implement near term applications [23,24]. Specifically, a *m* layered circuit $U\left(\theta \right)=\prod_{i=1}^{m}U_{i}\left(\theta_{i}\right)$ is presented in Figure 2a, which is parameterized by θ. As NISQ devices are with characteristics such as, limited size, short coherence time, and basic quantum operations, a shallow layered circuit seems perfect to undertake a NISQ applications. Remarkably, although shallow circuits and basic operations are utilized, the expressivity of layered circuits can be nontrivial and has been investigated in some recent papers [25,26]. Therefore, in our work, a layered circuit is employed to approach the simulation of matrix evolution, mathematically,

(3) $$\begin{array}{c} U_{m}\left(\theta_{m}\right)\ldots U_{i}\left(\theta_{1}\right)\to e^{-it\rho }, \end{array}$$

where ρ is target hermitian matrix.

A layered circuit can be fully determined by its structure and parameters. For most applications, structure is configured previously, which depends on the tasks at hand. The widely used structures include quantum alternating operator ansatz, variational Hamiltonian ansatz and unitary coupled clustered ansatz [27,28,29,30]. In this study, according to hardware efficiency, we employed an m=O(n) layered circuit for following *n*-qubit tasks. All two body interactions are involved and gates in one layer are commuted with each others, which aims at employing the two-body interactions of the devices with a limited depth circuit. This circuit is problem-agnostic, which is given in Appendix B For specific problem faced, the circuit should be re-designed, which exploits both expressibility and trainability.

Furthermore, before stepping into parameter determination, ancillary layered circuit is introduced as preliminary. It is schematically presented in Figure 2b. Besides the principal system which is denoted as *p*, the ancillary register *a* is added with the same size as Chio-Jamiolkowski isomorphism is employed. The entire circuit includes three parts: encoding circuit ($U_{e}$, colored blue), layered circuit part ($U_{l}⊗I$, colored pink), and decoding circuit ($U_{d}$, colored green). The details of dynamics in Figure 2b are shown as follows.

For initialization, two registers are jointly prepared on $\Omega_{0}=|\psi_{0}\rangle \langle \psi_{0}|$, where

(4) $$\begin{array}{c} |\psi_{0}\rangle ={|0\rangle }_{p}{|0\rangle }_{a}. \end{array}$$

Encoding circuit $U_{e}$ is supposed to evolve the system into $\Omega_{1}=|\psi_{1}\rangle \langle \psi_{1}|$, where $|\psi_{1}\rangle =\sum_{i}{|i\rangle }_{p}⊗{|i\rangle }_{a}$ are pairs of Bell states. This step can be realized by a bunch of control-*z* gates, $C_{z}$ and Hadamard gates, $H_{a}$,

(5) $$\begin{array}{ccc} \Omega_{1} & = & U_{e}\Omega_{0}U_{e}^{†},\\ U_{e} & = & I_{p}⊗H_{a}\cdot C_{z}\cdot H_{p}⊗H_{a}. \end{array}$$

Then, $U_{l}$ is applied on *p*, driving the system into $\Omega_{2}$, i.e., the Choi matrix of $U_{l}$,

(6) $$\begin{array}{c} \Omega_{2}=|\psi_{2}\rangle \langle \psi_{2}|=\sum_{i,j}U_{l}{\left(|i\rangle \langle j|\right)}_{p}U_{l}^{†}⊗|i\rangle {\langle j|}_{a}. \end{array}$$

Noted that, arbitrary state ρ with the application of $U_{l}$ has a state-channel duality,

(7) $$\begin{array}{c} U_{l}\left(\rho \right)={\mathrm{tr}}_{a}\left[\Omega_{2}\cdot I⊗\rho^{*}\right]. \end{array}$$

Thus, knowing $\Omega_{2}$ is sufficient to completely determine $U_{l}$, i.e., converting quantum channel characterization to state characterization. Choi-Jamiolkowski isomorphism is exactly the correspondences in Equations (6) and (7).

Finally, $\Omega_{3}$ is the result generated by steering $\Omega_{2}$ through the decoding circuit $U_{d}=U_{e}^{†}$, which disentangles the system and is essential in our methods.

For parameter determination, it can be solved by two optimization methods, which minimize the distance,

(8) $$\begin{array}{c} ∥U_{m}\left(\theta_{m}\right)\ldots U_{i}\left(\theta_{1}\right)-e^{-it\rho }∥, \end{array}$$

where ρ is the target hermitian matrix. Specifically, we substitute $U_{i}\left(\theta_{i}\right)$ with $e^{-it\theta_{i}\rho_{i}}$, where $\rho_{i}$ are tensor products of Pauli operators. In this configuration, the problem states as approaching $e^{-it\rho }$ by

(9) $$\begin{array}{c} e^{-it\theta_{m}\rho_{m}}\ldots e^{-it\theta_{1}\rho_{1}}. \end{array}$$

The formalism of $\rho_{i}$ determines the structure of layered circuit. It is an empirical task and is set in advance, which we have depicted before.

From the point of view of state, for a specified time *t*, ρ drives an ideal arbitrary system, which is labelled as σ, to

(10) $$\begin{array}{c} \sigma_{0}\left(t\right)=e^{-it\rho }\sigma e^{it\rho }. \end{array}$$

Simultaneously, with the application of Equation (9), the real system is steered to σ(t), where

(11) $$\begin{array}{c} \sigma \left(t\right)=\prod_{i=1}^{m}e^{-it\theta_{i}\rho_{i}}\sigma \prod_{i=1}^{m}e^{it\theta_{i}\rho_{i}}. \end{array}$$

The objective function *f* is thus defined as the overlap which is measured by the standard inner product

(12) $$\begin{array}{c} f\left(\theta \right)=\mathrm{tr}\left(\sigma_{0}\left(t\right)\sigma \left(t\right)\right)=\mathrm{tr}\left(\mu_{k}\cdot \nu_{k}\right), \end{array}$$

where

(13) $$\begin{array}{ccc} \mu_{k} & = & \prod_{i=k+1}^{m}e^{it\theta_{i}\rho_{i}}e^{-it\rho }\sigma e^{it\rho }\prod_{i=m}^{k+1}e^{-it\theta_{i}\rho_{i}},\\ \nu_{k} & = & \prod_{i=k}^{1}e^{-it\theta_{i}\rho_{i}}\sigma \prod_{i=1}^{k}e^{it\theta_{i}\rho_{i}}. \end{array}$$

Obviously, f(θ)≤1, the maximum situation is satisfied when the outputs of $\prod_{i=1}^{m}e^{-it\theta_{i}\rho_{i}}$ and $e^{-it\rho }$ are the same. This is a state to state situation. To approach the dynamics, Choi matrix is employed, which can be produced by ancillary layered circuit. In this situation, the notations are redefined,

(14) $$\begin{array}{ccc} \sigma & = & U_{e}\Omega_{0}U_{e}^{†},\\ \mu_{k} & = & \prod_{i=k+1}^{m}\left(e^{it\theta_{i}\rho_{i}}\cdot e^{-it\rho }\right)⊗I\cdot \sigma \cdot \prod_{i=m}^{k+1}\left(e^{it\rho }\cdot e^{-it\theta_{i}\rho_{i}}\right)⊗I,\\ \nu_{k} & = & \prod_{i=k}^{1}e^{-it\theta_{i}\rho_{i}}⊗I\cdot \sigma \cdot \prod_{i=1}^{k}e^{it\theta_{i}\rho_{i}}⊗I. \end{array}$$

*Method 1* is a traditional method of gradient, where the partial derivative with respect to the parameter $\theta_{k}$ are

(15) $$\begin{array}{c} \frac{\partial f}{\partial \theta_{k}}=-it\langle \mu_{k}|\left[\rho_{k},\nu_{k}\right]\rangle . \end{array}$$

〈·〉 means trace here. Therefore, by updating $\theta_{k}$ by $\theta_{k}^{\prime }$ with a learning rate η,

(16) $$\begin{array}{c} \theta_{k}^{\prime }=\theta_{k}+\eta \frac{\partial f}{\partial \theta_{k}}, \end{array}$$

the objective function would increase along the direction of gradient until converging into the local maximum. If f≈1 is iteratively achieved, this layered circuit is said to approach the application of $e^{-it\rho }$.

However, one vital problem is at calculating the gradient. As obtaining $\nu_{k}$ and $\mu_{k}$ is in general inefficient for tremendous time-consuming. Therefore, *Method 2* is reported.

*Method 2* is outlined as follows, (i) implementing $e^{i\rho \Delta t}$ with a layered circuit, where Δt is a sufficiently short period of time; (ii) implementing $e^{i\rho t}$ with (i) as the starting point step by step, where ancillary layered circuit is used as a compiler and O(log(t/Δt)) steps are required. This idea is extended from trotter decomposition, which induces a trotter error with $\epsilon_{t}∼O\left(t\Delta t\right)$. We illustrate the details as follows.

The first step is to realize an approximation,

(17) $$\begin{array}{c} \prod_{i}e^{-i\Delta t\theta_{i}\rho_{i}}\to e^{-i\Delta t\rho }, \end{array}$$

which requires us realizing following equation if only lowest order is considered,

(18) $$\begin{array}{c} \sum \theta_{i}\rho_{i}=\rho . \end{array}$$

This is coincident with Lie–Trotter decomposition, where sufficiently small Δt is adopted. Related details for derivation can be found in Appendix A. Accordingly, assuming that Δt is small enough, $e^{i\rho \Delta t}$ can be simulated efficiently by a layered circuit, where structure and parameters are given by Equation (18).

**Figure 3:**
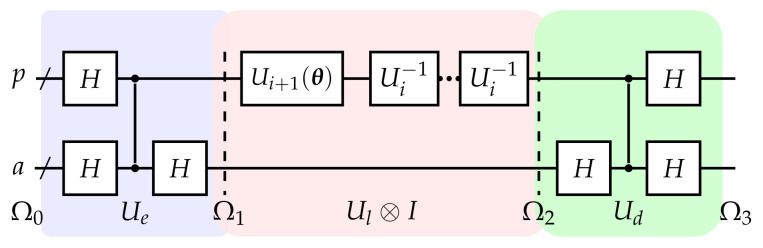
Ancillary layered circuit to implement *method 2*. $U_{i+1}\left(\theta \right)$ is the circuit to be optimized with known $U_{i}$. Notations are the same as Figure 2a. $\Omega_{0}$, $\Omega_{1}$, $\Omega_{2}$, and $\Omega_{3}$ are temporary states.

The second step is to simulate $e^{i\rho t}$. On basis of (i), this can be achieved by repeatedly applying ancillary layered circuit as compiler for O(log(t/Δt)) times. We depict the *i*-th iteration in Figure 3, where $U_{l}$ consists of $U_{i+1}\left(\theta \right)$ and $U_{i}^{-1}$ followed by $n_{c}$ times. The ancillary layered circuit serves as a compiler, learning repeated $U_{i}$ by $U_{i+1}\left(\theta \right)$. $n_{c}$ represents the compression efficiency, $U_{i}$ is known, and the structure of $U_{i+1}$ is set previously. Specifically, the task for *i*-th iteration is to generate $U_{i+1}\left(\theta \right)$ with an explicitly prior known $U_{i}$, where $U_{i+1}\leftarrow U_{i}^{n_{c}}$ and θ are the parameters to be optimized.

The objective function is defined as,

(19) $$\begin{array}{c} f=tr\left(\Omega_{1}\Omega_{2}\right)=tr\left(\Omega_{0}\Omega_{3}\right), \end{array}$$

which reaches the maximum at $U_{i+1}\left(\theta \right)=U_{i}^{n_{c}}$. As $\Omega_{1}$ are pairs of Bell states, this objective function cannot be measured with the local bases directly and efficiently. We investigate the second term in Equation (19): A decoding circuit $U_{d}$ disentangles the target component into $\Omega_{3}$. In this situation, only with the local measurement on $\Omega_{0}$, the objective function can be estimated and optimized as conventional hybrid quantum algorithms [31]. Iteratively, parameters in $U_{i+1}$, which are obtained in the *i*-th iteration, could be applied in the (i+1)-th step, where $U_{i+2}=U_{i+1}^{n_{c}}$ is realized.

Accordingly, by log(t/δ) steps, $e^{it\rho }$ can be implemented based on $e^{i\Delta t\rho }$. Compared to repeatedly applications of $U_{i}$ which will cause the circuit depth affordable, this strategy trades the circuit depth with repeated applications of ancillary layered circuit(shown in Figure 3), which increases the training time while compresses the repeated circuit depth.

## 3. Analysis

To simulate hermitian matrix evolution with layered circuit, time complexity is bounded by the depth of used circuit, which is easily analyzed if circuits are determined. For errors introduced by simulation, it is the distance between target evolution and layered circuit, which is also generated by two embedded methods. Therefore, analysis for embedded methods are important: Learning complexities and learning errors are analyzed in this section.

We list procedures of two methods in the *Method 1* and *Method 2* and analyze the learning complexity first.

*Method 1* relies on a classical optimization to generate parameter configurations. Therefore, complexities for time and memory is based on classical resources, such as classical logic gates and registers. During the procedure, approximating the gradient by Equation (15) costs the most. For each iteration, calculating $\mu_{k}$ and $\nu_{k}$ lies at the heart, which requires the implementation of matrix multiplications on a classical computer. Though $e^{-it\rho_{i}}$ can be efficiently simulated individually for locality of $\rho_{i}$, simulating $e^{-it\rho }$ is in general hard for most cases and there is no even universal efficient algorithms. To simulate an *n*-qubit problem, $2^{n}\times 2^{n}$ matrix should be generated and stored, with $O(4^{n})$ operations for evaluating the objective function and obtaining the gradient. Thus, the time complexity is $O(4^{n})\times r$ for a total *r* iterations while memory complexity is $O(4^{n})$. As Choi states are employed, our expedition will be enlarged, qubits are doubled.

Accordingly, this method is in general inefficient. However, it would be applicable to specific circumstances where $\nu_{k}$ and $\mu_{k}$ can be efficiently simulated. In fact, some investigations have employed tensor network to work out certain structure simulation problem and shed the light on the middle-scale quantum systems [32].

*Method 2* is a hybrid quantum classical paradigm, where ancillary layered circuits are executed on quantum computers, parameters are updated on classical computers. For tasks on quantum computers, ancillary layered circuit brings a 2n-qubit consumption on quantum register, where *n* is the size of simulation quantum system. Time complexity to execute ancillary layered circuit is additional O(n) Hadamard and control-z gates, with a depth of O(m) layered circuit consisting of at most O(m×n) basic quantum gates. Luckily, our measurement is on $\Omega_{0}$, which is local measurement. Therefore, for single iteration, updating all parameters requires O(m×n) times repeatedly applying and measure ancillary layered circuit. For *r* repetitions, O(m×n)×r is required. If $e^{-it\rho }$ is to be realized by $e^{-i\Delta t\rho }$, an additional multiplied factor log(t/Δt) should be added. For tasks on classical computers, a storage of O(m×n) parameters and their numerical gradient is required. Time complexity depends only on operations of fundamental arithmetic, which is within the reach of current machines.

Accordingly, time complexity of *Method 2* is O(m×n×r×log(t/Δt)). As in our configuration, m=O(n), which leads our method acceptable with respect to efficiency.


**Method 1**
  **Input:** layered circuit $\prod_{i=1}e^{-it\theta_{i}\rho_{i}}$. θ is to be optimized, $\epsilon_{o}$ is an optimized threshold, and $\delta_{1}$ is the tolerance for improving.  **Output:**
θ, parameter configuration, which is optimized to approach $e^{-it\rho }$ via $\prod_{i=1}e^{-it\theta_{i}\rho_{i}}$. 1: Evaluate the objective function Equation (12) with existed θ, denoted as *f*. If $f\leq 1-\epsilon_{o}$, go to 2, otherwise, the algorithm terminates and θ return.2: **for** 
k=1,…,m **do**3:   Based on Equation (14), $\nu_{k}$ and $\mu_{k}$ are calculated.4:   Evaluate $\partial f/\partial \theta_{k}$ by Equation (15).5: **end for**6: Update *m*-element θ according to Equation (16)7: Evaluate the objective function with the new θ8: **if**
$f\geq 1-\epsilon_{o}$
**then**
9:   the algorithm terminates and θ return;10:** else if**
$\Delta f\leq \delta_{1}$
**then**11:   θ are re-initialized and go to 1.12: **else**13:   go to 2.14: **end if**15:16:17:18:


**Method 2**
  **Input:** layered circuit $\prod_{i=1}e^{-i\theta_{i}\rho_{i}}$ with known $U_{1}$. θ is to be optimized, $\epsilon_{o}$ is the optimized threshold, $\delta_{2}$ is the tolerance for improving, and $n_{c}$ is the compressing factor.  **Output:**
θ, parameter configuration, which is optimized to approach $e^{-it\rho }$ via $\prod_{i=1}e^{-it\theta_{i}\rho_{i}}$ 1: **for** i=1,…,log(t/Δt) **do**2:   **if**
i=1
**then**3:    $U_{1}$ and its inversion are generated by the input.4:   **else**5:    $U_{i}$ are generated as the output of the last iteration.6:   **end if**7:   Evaluate the objective function in Equation (19) with existed θ and $n_{c}$, which is denoted as *f*. If $f\leq 1-\epsilon_{o}$, go to 8, otherwise, θ for $U_{i+1}$ return and go to 1.8:   θ for $U_{i+1}$ are optimized as variant quantum algorithms, θ are updated and *f* is evaluated.9:   **if**
$f\geq 1-\epsilon_{o}$
**then**10:    θ return and go to 1;11:   **else if**
$\Delta f\leq \delta_{2}$
**then**12:    θ are re-initialized and go to 7.13:   **else**14:    go to 8.15:   **end if**16: **end for**

To analyze errors of layered circuit by both methods, first, we define some notations: $\epsilon_{o}$ is the optimized threshold which is supposed to terminate the training process; $\epsilon_{t}$ is the deviations coming from lie-product decomposition.

If *Method 1* is completed, the optimized threshold is targeted. The obtained layered circuit has an accuracy of $1-\epsilon_{o}$ as training process permits the error no more than $\epsilon_{o}$. For *Method 2*, the error accumulates according to a chain rule when implementing $U_{i+1}$ by $U_{i}$. It ends up with an error of $O(n_{c}^{s}\epsilon_{o})$, where the higher order terms are ignored and *s* is steps, which is in total of O(log(t/Δt)) to realizing $e^{-i\rho t}$ from $e^{-i\rho \Delta t}$. Thus, the discrepancy is $O(\left(t/\Delta t\right)\epsilon_{o})$. Additionally, an error coming from Equation (1) is also considered as $\epsilon_{t}$, which is of O(tΔt). Accordingly, the total error for *Method 2* is $O\left(\left(t^{2}/\epsilon_{t}\right)\epsilon_{o}\right)+O\left(\epsilon_{t}\right)$.

In the end of this section, we analyze the expressive power for typical layered circuit, which is given by our appendix method. In fact, it is problem-dependent and can be replaced with smarter ansatz with tasks at hand.

**Figure 4:**
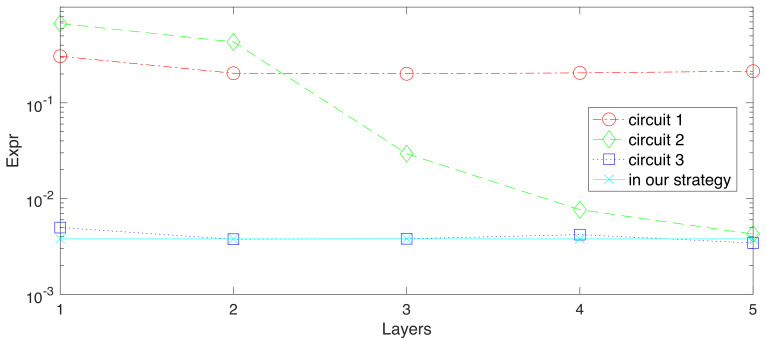
Expressibility values computed for circuits specified in Appendix C and circuit used in our protocol. Circuit 1 (red circles), 2 (green diamonds) and 3 (blue squares) are repeatedly applied with up to 5, while the circuit employed in our strategy (cyan cross) keeps unchanged.

**Figure A1:**
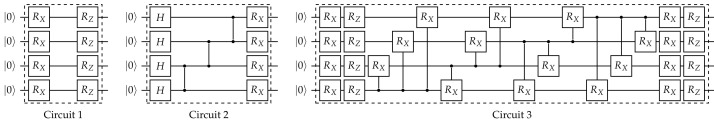
A set of circuit templates considered in the study, each labeled with a circuit ID. The dashed box indicates a single circuit layer, denoted by *L* in the text, that can be repeated.

Expressibility is proposed recently as a distance, which measures the output states distribution of layered circuits and the Haar [26]. To explicitly present this value, Kullback-Leibler (KL) divergence(or relative entropy) is employed to estimate this distance, which is denoted as *Expr*. A highly expressible circuit would produce a small Kullback-Leibler value. In this part, besides numerically calculating Expr of circuit given in Appendix C, three other types of parametrized circuits are also studied as comparisons, which are also specified in Appendix C. Three compared circuits are repeated with up to 5 times to calculate Expr, while our typical circuit stays unchanged. Figure 4 shows the results of Expressibility values (or KL divergences), where circle, diamond and square represents three comparison circuits and cyan cross labels the circuit given by us. The layered circuit in this work has a similar performance as multi-applications of circuit 2 and 3 in Figure A1. Remarkably, in general, repeating a circuit layer would increase the expressive power. However, as no entanglement gate exists in circuit 1 in Figure A1, this argument does not hold for that circuit. More information on expressibility and its simulation can be found in Appendix C or related work [26].

## 4. Numerical Experiments

In this section, numerical experiments are investigated, including applications on Hamiltonian simulation and density matrix evolution. In our protocol, ρ is taken as (a) Bell state; (b) GHZ state and (c) Hamiltonian of a liquid NMR sample, Crotonic acid, which is specified in the Appendix E.

To generate the simulating layered circuit, two learning methods are employed, with

(20) $$\begin{array}{c} f\left(\theta \right)=tr\left(\rho_{t}\Omega_{3}\right) \end{array}$$

being the objective function. θ=α,β are for *Method 1* and *Method 2*. The target state can be expressed as,

$$\begin{array}{ccc} & \rho_{t}=U_{d}\cdot e^{-i\rho t}⊗I_{a}\cdot U_{e}\left(\Omega_{0}\right)\; & \left(Method\;1\right),\\ & \rho_{t}=\Omega_{0}\;\; & \left(Method\;2\right). \end{array}$$

And additionally, $\Omega_{3}=U_{d}\cdot U⊗I_{a}\cdot U_{e}\left(\Omega_{0}\right)$, where

$$\begin{array}{ccc} & U=U_{1}\left(\alpha \right)=\prod_{i=1}^{m}e^{-it\alpha_{i}\rho_{i}}\; & \left(Method\;1\right),\\ & U=U_{2}\left(\beta \right)=U_{i}^{-n_{c}}U_{i+1}\left(\beta \right)\; & \left(Method\;2\right). \end{array}$$

This is specified in Appendix D.

To find optimal parameter configuration, gradient-based method is employed. To begin with, random numbers by a single uniformly distribution are generated and assigned as the value of initial parameters. During the training process, the approximation of the partial derivatives can be estimated by following symmetric difference quotient

(21) $$\begin{array}{c} \partial_{j}f=\frac{f\left(\theta +\Delta \theta_{j}\right)-f\left(\theta -\Delta \theta_{j}\right)}{2\Delta \theta_{j}}, \end{array}$$

which can be realized by repeatedly running the training circuit with a small perturbation on the θ, where $\Delta \theta_{j}$ is naively chosen as 0.01 in our simulation. Therefore, the optimal configuration of the parameters can be obtained by repeating

(22) $$\begin{array}{c} \theta =\theta +\eta \nabla f(\theta ), \end{array}$$

where $\nabla f\left(x\right)=\left(\partial_{1}f,\partial_{2}f,\ldots \right)$ and the learning rate, η, is fixed at 0.02. For simulating *Method 1* and *Method 2*, the maximum numbers for repeating Equation (22), i.e., iterations are set as 300 and 350, respectively.

In this prototypical simulation, the program is conducted with assumptions that all one or two quantum gates are accurate. Three circuits with the depths of 3, 4 and 5 are employed for the simulation the evolution. The numbers of parameters to be optimized are thus 12, 24 and 40, which converge to O(m×n), where *m* is the depth and *n* is the size of circuit. As the comparison, if ϵ is tolerant error, O(3/ϵ), O(4/ϵ) and O(5/ϵ) depth circuits are required for simulation by lie-product decomposition. For density matrix evolution, O(1/ϵ) copies of density matrix are required as with O(1/ϵ)-depth circuit.

For data collection, strategies are different for *Method 1* and *Method 2*. For *Method 1*, once the optimization completed, parameters is supposed to be optimal for simulating a *t*-time interval evolution. Otherwise, we need to re-run the simulation and repeat Equation (22) until the objective function satisfying the optimized threshold. For *Method 2*, optimizing ancillary layered circuit is not once for all. After the *i*-th repetition of training ancillary layered circuit with Equation (22) for 350 iterations, only $U_{i}^{-n_{c}}U_{i+1}\left(\beta \right)$ is learned. Therefore, O(log(t/Δt)) repetitions are required. In our simulation, $\Delta t/t=1/2^{10}$, i.e., 10 steps are required and the base number, 2, determines the compression efficiency of the circuit.

**Figure 5:**
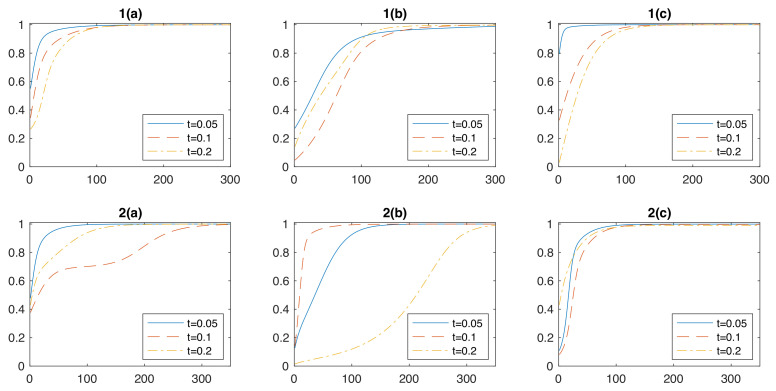
Results of *Method 1* and *Method 2* for simulating the evolutions by ρ for a period of t=0.05, 0.1 and 0.2. The fidelities (vertical axis) vary with the iterations (horizon axis). ρ is chosen as (**a**) bell state; (**b**) GHZ state and (**c**) Hamiltonian of a molecular Crotonic acid system.

Figure 5 shows the results of our simulation, which provide supports on our protocol by converging to the target matrix evolutions. (a) (b) and (c) simulate the evolutions of Bell state, GHZ state and Hamiltonian of Crotonic acid, where the evolution timescales are set as 0.05, 0.1 and 0.2, respectively. Fidelities are shown by vertical axis and calculated by tracing the inner product of two operators. The results show that, with the accurate local quantum gate assumption, the simulating circuit gets more and more like the evolution of certain matrix evolution as with the number of iterations.

## 5. Limitation

In the end, defects in our protocol should be listed. First, only Hermitian operations can be dealt with. Evolution of an open quantum system is not considered in this article. With formalism for the description of open quantum systems, this target is potentially solved in future work [33]. Second, similar to the widely-used parameterized circuits architecture such as QAOA and VQE, expressive ability of ansatz in our methods determinate the upper-bound of accuracy [25,28]. Therefore, a proper ansatz will have a good performance. If the termination threshold cannot be reached, an alternative ansatz or iterative training algorithms should be resorted to. Third, optimization should be clarified, which is also the most important one. Many optimization problems are in fact NP-hard problems. The methods embedded are essentially optimization-based, and cannot get over them, too. Taking an example of gradient-based methods, they cannot avoid the local optimum problems, especially when the feasible region is complicated. Therefore, the proposed methods would fail in finding a global optimum with a bad initial guess, just as the classical cases. Although numerical results are good without considering the initialization, we have to admit a good initial guess is crucial to decrease the possibility of failure, especially for dealing with a larger problem. Investigations on the optimization method itself should attract more attention, which benefits not only the exploitation of near-future quantum devices but also for most modern technologies [34,35].

## 6. Conclusions

NISQ era has come in and would last for decades. Thus, finding a near-future algorithm which exploits the power of NISQ devices becomes more and more important. Simulating matrix evolution, which is an essential module for many quantum algorithms, such as density matrix evolution for quantum principal component analysis, and static or dynamic simulation for quantum systems. The existed implementation still relies on a deep quantum circuit, which is intractable with current NISQ devices.

In this paper, a heuristic layered circuit protocol is proposed for simulating $e^{-i\rho t}$ with Hermitian ρ. To construct these circuits, two methods are given. *Method 1* is with the classical optimal control theory and *Method 2* is with the hybrid quantum-classical paradigm. For *Method 1*, learning a layered circuit requires $O(4^{n}\times r)$ operations, where *n* is the size of quantum circuit and *r* is the number of total iterations. As computational resource may be put into its limits, we provide *Method 2*, which is hybridized with a quantum agent. As a comparison, time complexity is O(m×n×r×log(t/Δt)), with 2n being the size of circuit and *m* being circuit depth. From the point of view of error, although *Method 1* is in general inefficient, the error can be bounded to $O(\epsilon_{o})$. *Method 2* introduces a larger one, $O\left(\left(t^{2}/\epsilon_{t}\right)\epsilon_{o}\right)+O\left(\epsilon_{t}\right)$. Only when $\epsilon_{o}$ is sufficiently small, *Method 2* can approach an accuracy as sTrotter method. Accordingly, circuits by both methods would be shallow, which is easily deployed on current devices.

Simulating matrix evolution is important for many quantum information tasks. Our protocol, which realizes hermitian matrix evolutions, is supposed to contribute to the field of NISQ algorithm. For layered circuit in our both methods, only basic quantum gates are required, which means at most 2-qubit interactions are needed. With respect to experimental technology, it is already mature for most physical platforms, such as optical lattice, spin-based, and superconducting qubit [7,36,37]. In despite of some limitations, the protocol is with an affordable computational consumption, which paves a way for possibly applications on NISQ devices. In addition, ancillary layered circuit, which serves as a compiler in this article, is promising to be an important subroutine in near future and provides a new way to exploit the power of NISQ devices.

## Figures and Tables

**Figure A2:**
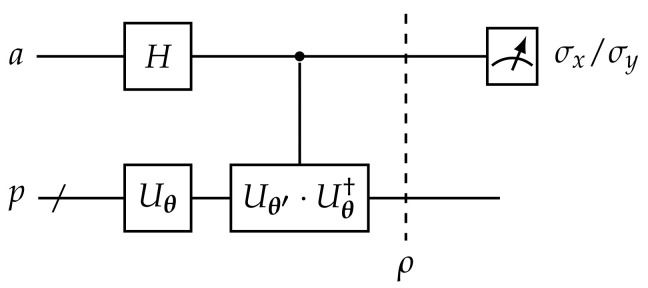
Circuit for measurement of the expressivity of the layered circuit. It costs one extra qubit as ancillary. θ and $\theta^{\prime }$ are two random parameter configurations for layered circuits $U_{\theta }$ and $U_{\theta^{\prime }}$, *H* is a one-qubit Hadamard gate.

**Figure A3:**
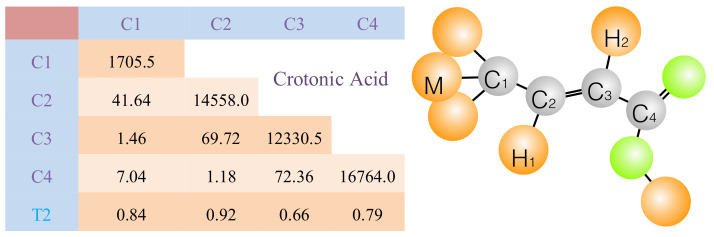
Molecular structure of ${}^{13}$C-labeled Crotonic acid. $\nu_{j}$ and $J_{jk}$ are the chemical shifts and J-couplings, respectively, which are listed by the diagonal and off-diagonal elements. T${}_{2}$ (in Seconds) are the relaxation time which are shown at bottom.

## Data Availability

All data for the figure and table are available on request. All other data about experiments are available upon reasonable request.

## Abbreviations

The following abbreviations are used in this manuscript:

NISQ	Noisy Intermediate-Scale Quantum

## Appendix A. Derivations on $e^{-i\Delta t\rho }$ in *Method 2*

First, if one wants to simulate $e^{-i\Delta t\rho }$ with $\prod_{i}e^{-i\Delta t\beta_{i}\rho_{i}}$, the idea of optimizations can always be resorted to. An objective function can thus be defined as,

(A1) $$\begin{array}{c} f\left(\beta \right)=tr\left(e^{-i\Delta t\rho }\sigma e^{i\Delta t\rho }\prod_{i}e^{-i\Delta t\beta_{i}\rho_{i}}\sigma \prod_{i}e^{i\Delta t\beta_{i}\rho_{i}}\right), \end{array}$$

where σ is an arbitrary initial density matrix, and operators in 〈·〉 satisfy the permutation equality. We expand matrix exponentiation in Equation (A1) with Taylor series on Δt,

(A2) $$\begin{array}{ccc} e^{-i\Delta t\rho } & = & \sum_{k=0}^{\infty }{\left(-i\Delta t\rho \right)}^{k}/k!,\\ \prod_{i=1}^{N}e^{-i\Delta t\beta_{i}\rho_{i}} & = & \prod_{i=1}^{N}\sum_{k=0}^{\infty }{\left(-i\Delta t\beta_{i}\rho_{i}\right)}^{k}/k!. \end{array}$$

With substitutions such as, H→−iΔtρ and $M_{i}\to -i\Delta t\beta_{i}\rho_{i}$, ingredients in Equation (A1) can be rewritten as,

$$\begin{array}{ccc} & & e^{i\Delta t\rho }\times \prod_{i=N}^{1}e^{-i\Delta t\beta_{i}\rho_{i}}\\ & & =\left(\frac{{\left(-H\right)}^{0}}{0!}+\frac{{\left(-H\right)}^{2}}{1!}+\frac{{\left(-H\right)}^{3}}{3!}+\cdots \right)\times \left(\frac{M_{N}^{0}}{0!}+\frac{M_{N}^{1}}{1!}+\frac{M_{N}^{2}}{2!}+\cdots \right)\ldots \left(\frac{M_{1}^{0}}{0!}+\frac{M_{1}^{1}}{1!}+\frac{M_{1}^{2}}{2!}+\cdots \right)\\ & & =\sum_{k=0}^{\infty }\sum_{\begin{array}{c} j_{i},j_{\rho }\geq 0,\\ \sum_{i}j_{i}+j_{\rho }=k \end{array}}\frac{{\left(-H\right)}^{j_{\rho }}}{j_{\rho }!}\prod_{i=N}^{1}\frac{M_{i}^{j_{i}}}{j_{i}!},\\ & & \prod_{i=1}^{N}e^{i\Delta t\beta_{i}\rho_{i}}\times e^{-i\Delta t\rho }\\ & & =\left(\frac{{\left(-M_{1}\right)}^{0}}{0!}+\frac{{\left(-M_{1}\right)}^{1}}{1!}+\frac{{\left(-M_{1}\right)}^{2}}{2!}+\cdots \right)\cdots \left(\frac{{\left(-M_{N}\right)}^{0}}{0!}+\frac{{\left(-M_{N}\right)}^{1}}{1!}+\frac{{\left(-M_{N}\right)}^{2}}{2!}+\cdots \right)\times \left(\frac{H^{0}}{0!}+\frac{H^{2}}{1!}+\frac{H^{3}}{3!}+\cdots \right)\\ & & =\sum_{k=0}^{\infty }\sum_{\begin{array}{c} j_{i},j_{\rho }\geq 0,\\ \sum_{i}j_{i}+j_{\rho }=k \end{array}}\prod_{i=1}^{N}\frac{{\left(-M_{i}\right)}^{j_{i}}}{j_{i}!}\frac{H^{j_{\rho }}}{j_{\rho }!}. \end{array}$$

Under the circumstances that no approximation is adopted, the objective function is

(A3) $$f=\langle \psi |\sum_{k=0}^{\infty }\sum_{\begin{array}{c} j_{i},j_{\rho }\geq 0,\\ \sum_{i}j_{i}+j_{\rho }=k \end{array}}\prod_{i=1}^{N}\frac{{\left(-M_{i}\right)}^{j_{i}}}{j_{i}!}\frac{H^{j_{\rho }}}{j_{\rho }!}\left|\psi \rangle \langle \psi \right|\sum_{k=0}^{\infty }\sum_{\begin{array}{c} j_{i},j_{\rho }\geq 0,\\ \sum_{i}j_{i}+j_{\rho }=k \end{array}}\frac{{\left(-H\right)}^{j_{\rho }}}{j_{\rho }!}\prod_{i=N}^{1}\frac{M_{i}^{j_{i}}}{j_{i}!}|\psi \rangle ,$$

where σ=|ψ〉〈ψ| and 〈·〉 represents the expectation on |ψ〉. Obviously, it is a taylor-like polynomial with respect to Δt. As Δt can be a sufficiently small quantity when we simulate $e^{-it\rho }$, only lowest order terms on Δt are considered. For the objective function *f*, all terms which is linear with or higher than $O(\Delta t^{2})$ are ignored. Therefore, *f* can be simplified as,

(A4) $$\begin{array}{c} {\left(1+<\frac{H^{2}}{2!}>-<H\sum_{i}M_{i}>+<\sum_{i}\frac{M_{i}^{2}}{2!}>+<\sum_{i>j}M_{i}M_{j}>\right)}^{2}-{\left(<H>-<\sum M_{i}>\right)}^{2}, \end{array}$$

where $\sum M_{i}=H$ is the requirement for eliminating above lowest order error, which is also independent of |ψ〉. In the original representation, $\beta_{i}$ satisfy following correspondence

(A5) $$\begin{array}{c} \sum \beta_{i}\rho_{i}=\rho . \end{array}$$

which is also the correspondence by Lie–Trotter products. Therefore, a parameterized quantum circuit for $e^{-i\Delta t\rho }$ can be obtained by Equation (A5), where $\rho_{i}$ is comparably easy-access to realize and benchmark in near-future quantum devices.

## Appendix B. Method to Generate an N-Layer Circuit

In this section, a method to generate an *N*-layer circuit structure with all two-body interactions is introduced, where *N* is the size of the system. $P_{2}\left(i,j\right)$ are assumed as the products of Pauli matrices, where $P_{2}\left(i,j\right)=\sigma_{k}⊗\sigma_{l}$ are on both *i*-th and *j*-th qubits and i,j=1,…,N and k,l=x,y,z. All of them can be presented in an N×N triangle parameter matrix *P*,   

(A6) $$\begin{array}{c} P=\left[\begin{array}{ccccccc} 1 & P_{2}\left(1,2\right) & P_{2}\left(1,3\right) & P_{2}\left(1,4\right) & P_{2}\left(1,5\right) & ,\cdots , & P_{2}\left(1,N\right)\\ & 2 & P_{2}\left(2,3\right) & P_{2}\left(2,4\right) & P_{2}\left(2,5\right) & ,\cdots , & P_{2}\left(2,N\right)\\ & & 3 & P_{2}\left(3,4\right) & P_{2}\left(3,5\right) & ,\cdots , & P_{2}\left(3,N\right)\\ & & & 4 & P_{2}\left(4,5\right) & ,\cdots , & P_{2}\left(3,N\right)\\ & & & & & ,\cdots , & \\ & & & & & & P_{2}\left(N-1,N\right)\\ & & & & & & N \end{array}\right], \end{array}$$

where $P_{ii}=i$, $P_{ij}=P_{2}\left(i,j\right)$ for i<j and $P_{ij}=0$ for i>j. Thus, the non-zeros elements in *P* can be divided into *N* group with the following Method A1, where *N* is supposed to be odd.

**Method A1** Algorithm on grouping the Pauli words
**Input:** Parameter matrix *P*, *N* null sets, and a N×N label matrix *L*, where $L_{mn}=1$ for arbitrary m,n. **Output:***N* sets of Pauli words:$S_{1},S_{2}\ldots ,S_{N}$ **for** i=1,…N **do** **for** j=i…,N **do** **if** $i=j\cap L_{ij}=1$ **then** $P_{ij}\in S_{i}$, $L_{ij}=0$. **else** **for** m=1…,N **do** **if** $L_{mi}=1\cap L_{mj}=1$ **then** $P_{ij}\in S_{m}$, $L_{mi}=0$, $L_{mj}=0$ **end if** **end for** **end if** **end for** **end for**

With the complexity of $O(N^{3})$ operations, Those Pauli words are divided into *N* group, where the Pauli words in each group commute with each other. Via matrix exponentiation formation multiplied by tunable parameters, they can be arranged into a parameterized circuit, with a depth of *N*, being linear with the size of the system. If different type Pauli combinations are considered, O(N) is the depth. Therefore, this algorithm provided a depth of O(N) parameterized circuit with all two-body interactions considered. Remarkably, this is one type of problem-agnostic circuit, which exploit the power of current quantum devices with two-body interactions available. For different problems, problem-inspired circuit can be designed, employing information about the problem, which bring better expressibility and trainability. Surely, we will study the expressibility of our proposed circuit in next section.

## Appendix C. Expressibility of the Layered Circuit

A layered circuit with excellent expression is more likely to represent a target state. To measure the expressive power of a circuit, expressibility is defined in recent work as a distance of two state distributions, characterized with ||·||, the Hilbert-Schmidt norm [26].

(A7) $$\begin{array}{c} A=\left|\right|\int_{Haar}{\left(|\psi \rangle \langle \psi |\right)}^{⊗t}d\psi -\int_{\theta }{\left(|\psi_{\theta }\rangle \langle \psi_{\theta }|\right)}^{⊗t}d\psi_{\theta }\left|\right|, \end{array}$$

where ${|\psi \rangle }_{\theta }$ are outputs of a layered circuit with randomized configuration θ and |ψ〉 are from a distribution according to the Haar measure. It is a generalization from the investigation of pseudo-random circuit. Thus, a highly expressible circuit would produce a small *A*, with A=0 corresponding to being maximally expressive, i.e., generating a state distribution to the Haar measure.

In order to explicitly estimate the expressibility with a discrete simulated result, the Kullback-Leibler (KL) divergence, i.e., relative entropy is employed, which measures the difference between one probability distribution and a reference probability distribution. It is denoted as Expr,

(A8) $$\begin{array}{c} Expr=D_{KL}\left(P_{l}\left(f|\theta ,\theta^{\prime }\right)|P_{Haar}\left(f\right)\right), \end{array}$$

where $P_{l}\left(f|\theta ,\theta^{\prime }\right)$ is the probability distribution of *f*=$|\langle \psi_{\theta }|\psi_{\theta^{\prime }}{\rangle |}^{2}$, outputs of a layered circuit with random θ and $\theta^{\prime }$. For the reference probability distribution, i.e., to Haar measure, $P_{Haar}\left(f\right)=\left(N-1\right){\left(1-f\right)}^{N-2}$ is analytical, with *N* being size of dimension. Expr scores the similarity to the distribution of Haar as a layered circuit with a lower value of KL divergence is a more expressible circuit.

Accordingly, numerical experiments to compute and compare the expressibility in the strategy with three other typical layered circuits are studied, which are shown in Figure A1. They are all 4 qubit circuit. As with the original work, to construct a distribution of $P_{l}$ with histogram, a bin size is set as 75, and 5000 *f* are measured. For each type of layered circuits except for the one in our strategy, the instances where the number of layers are investigated with up to 5. Compared with 10,000 times repeatedly running the circuits and state tomography, *f* can be measured directly with the setup proposed in Figure A2 for 5000 time and without tomography. Only 1-qubit is added as the ancillary system *A*, at the end of circuit, the system is evolved as

(A9) $$\begin{array}{c} \rho =|0\rangle \langle 0|⊗|\psi_{\theta }\rangle \langle \psi_{\theta }|+|1\rangle \langle 0|⊗|\psi_{\theta^{\prime }}\rangle \langle \psi_{\theta }|+|0\rangle \langle 1|⊗|\psi_{\theta }\rangle \langle \psi_{\theta^{\prime }}|+|1\rangle \langle 1|⊗|\psi_{\theta^{\prime }}\rangle \langle \psi_{\theta^{\prime }}|. \end{array}$$

Via the measurements on ancillary system, *f* can be obtained with the real and imaginary part of $\langle \langle \psi_{\theta }|\psi_{\theta^{\prime }}\rangle \rangle$, which are extracted as

(A10) Re=tr(σx⊗I·ρ)=12(〈ψθ|ψθ′〉+〈ψθ′|ψθ〉)Im=tr(σy⊗I·ρ)=−i2(〈ψθ|ψθ′〉−〈ψθ′|ψθ〉).

Results are shown in the Figure 4 in main text. For three types of layered circuits which are denoted in circle, diamond and square, the instances are considered where the circuit, which is as a unit layer, are repeated with up to 5. For the circuit proposed by Method A1, the repetition is always 1.

In general, repeating a circuit layer in Figure A1 would increase the expressibility. As there is no entanglement gate in circuit 1, this argument does not hold. For circuit 2 and 3, even if a single layer for circuit 3 have pretty good expression, this argument holds. From the result, there are convergences for the metric of expressibility. For the argument whether more numbers of layers can help the expression, at least, we can not obtain more information from this metric. Additionally, the layered circuit generated in our strategy, which is labeled as cyan cross, keeps a same structure. It has a similar performance with respect to the repeated circuits 2 and 3. As a small value of expressibility imply an excellent expression, the generated circuits in the strategy performs no worse than other typical circuit.

## Appendix D. Objective Functions

In this section, the explicit expression of the objective functions is derived, as well as the step-by-step illustrations of the circuit to implement the Numerical simulation. The objective function can be concluded as

(A11) $$\begin{array}{c} f\left(\theta \right)=tr\left(\rho_{t}\Omega_{3}\right), \end{array}$$

where θ=α,β are for *Method 1* and *Method 2*, respectively. As for intermediate states and quantum operations of the circuit shown in original manuscript, they are depicted as,

(A12) $$\begin{array}{ccc} \Omega_{0} & = & |\psi_{0}\rangle \langle \psi_{0}|,\;|\psi_{0}\rangle ={|0\rangle }_{p}{|0\rangle }_{a},\\ \Omega_{1} & = & |\psi_{1}\rangle \langle \psi_{1}|,\;|\psi_{1}\rangle =U_{e}|\psi_{0}\rangle ,\\ \Omega_{2} & = & |\psi_{2}\rangle \langle \psi_{2}|,\;|\psi_{2}\rangle =U_{l}⊗I_{a}|\psi_{1}\rangle ,\\ \Omega_{3} & = & |\psi_{3}\rangle \langle \psi_{3}|,\;|\psi_{3}\rangle =U_{d}|\psi_{2}\rangle . \end{array}$$

where $U_{e}=I_{p}⊗H_{a}\cdot C_{z}\cdot H_{p}⊗H_{a}$, $U_{d}=H_{p}⊗H_{a}\cdot C_{z}\cdot I_{p}⊗H_{a}$. In addition, $|\psi_{1}\rangle =\sum_{i=1}^{d}{|i\rangle }_{p}⊗{|i\rangle }_{a}$ is the bell state pairs, where the idea of Choi matrix for assisting tomography is employed.

With respect to the $U_{l}$ in *Method 1*, it aims at finding optimal parameters $\alpha =(\alpha_{1},\alpha_{2},\ldots )$ to generate a layered circuit. While, for the *i*-th repetition of *Method 2*, it aims at finding appropriate parameters $\beta =(\beta_{1},\beta_{2},\ldots )$, generating a layered circuit which realizes repeatedly applied $U_{i}$. Thus, the different $U_{l}$ are to be optimized

$$\begin{array}{ccc} & U_{1}\left(\alpha \right)=\prod_{i=1}^{m}e^{-it\alpha_{i}\rho_{i}}\; & \left(Method\;1\right),\\ & U_{2}\left(\beta \right)=U_{i}^{-2}U_{i+1}\left(\beta \right)\; & \left(Method\;2\right). \end{array}$$

As for target states, they are also different. *Method 1* realizes an evolution by a target Hermitian matrix on system *p* while *Method 2* realizes $I_{p}$. Therefore, the density matrix $\rho_{t}$ for both strategies are

$$\begin{array}{ccc} & \rho_{t}=U_{d}\cdot e^{-i\rho t}⊗I_{a}\cdot U_{e}\left(\Omega_{0}\right)\; & \left(Method\;1\right),\\ & \rho_{t}=\Omega_{0}\;\; & \left(Method\;2\right), \end{array}$$

where ρ can be density matrix or Hamiltonian, for simulating hermitian matrix exponentiation.

## Appendix E. Matrices for Numerical Simulation

In this section, the detailed information for ρ in numerical simulation are given. Three cases are studied. Two of them are density matrices and one is a Hamiltonian from previous experiments. The specified form are as follows.

*a. Bell State*

(A13)
$$\begin{array}{c} |\psi \rangle =\frac{1}{\sqrt{2}}\left(|00\rangle +|11\rangle \right),\;\rho =\frac{1}{2}\left(\begin{array}{cccc} 1 & 0 & 0 & 1\\ 0 & 0 & 0 & 0\\ 0 & 0 & 0 & 0\\ 1 & 0 & 0 & 1 \end{array}\right). \end{array}$$

*b. GHZ State*

(A14)
$$\begin{array}{c} |\psi \rangle =\frac{1}{\sqrt{2}}\left(|000\rangle +|111\rangle \right),\;\rho =\frac{1}{2}\left(\begin{array}{cccccccc} 1 & 0 & 0 & 0 & 0 & 0 & 0 & 1\\ 0 & 0 & 0 & 0 & 0 & 0 & 0 & 0\\ 0 & 0 & 0 & 0 & 0 & 0 & 0 & 0\\ 0 & 0 & 0 & 0 & 0 & 0 & 0 & 0\\ 0 & 0 & 0 & 0 & 0 & 0 & 0 & 0\\ 0 & 0 & 0 & 0 & 0 & 0 & 0 & 0\\ 0 & 0 & 0 & 0 & 0 & 0 & 0 & 0\\ 1 & 0 & 0 & 0 & 0 & 0 & 0 & 1 \end{array}\right). \end{array}$$

*c. Hamiltonian of Crotonic Acid*

The Hamiltonian simulated in our numerical experiments is from ${}^{13}$C-labeled Crotonic acid dissolved in d6-acetone, which is usually employed as a four-qubit quantum system for NMR-based quantum information processing [18].

The structure of this molecule is shown in Figure A3, where C${}_{1}$ to C${}_{4}$ are four carbon atoms, *M* is one of three hydrogen atoms in a methyl group, H${}_{1}$ and H${}_{2}$ are two hydrogen atoms. When it is used as a four qubit quantum system, the hydrogen atoms are decoupled from the carbon atoms with a shaped radio frequency pulse. Under the circumstance of weak coupling, the internal Hamiltonian of this molecule can be expressed as,

(A15) $$\begin{array}{c} H_{int}=\sum_{j=1}^{4}\frac{1}{2}\nu_{j}\sigma_{z}^{j}+\sum_{j<k}^{4}\frac{\pi }{2}J_{jk}\sigma_{z}^{j}\sigma_{z}^{k}. \end{array}$$

$\nu_{j}$ and $J_{jk}$ are inside Hamiltonian parameters whose values are listed in the table besides the molecule. $\nu_{j}$, the chemical shift, are the diagonal elements and $J_{jk}$, the J-coupling, are the off-diagonal elements. As for $T_{2}$, the transverse relaxation time, is not involved in our simulation as the unitary process is assumed. All parameters can be found in related paper which is under a magnetic field of 9.4 T at room temperature (296.5 K).
